# The clinical significance of sub-total surgical resection in childhood medulloblastoma: a multi-cohort analysis of 1100 patients

**DOI:** 10.1016/j.eclinm.2024.102469

**Published:** 2024-02-14

**Authors:** Claire Keeling, Simon Davies, Jack Goddard, Vijay Ramaswamy, Edward C. Schwalbe, Simon Bailey, Debbie Hicks, Steven C. Clifford

**Affiliations:** a Wolfson Childhood Cancer Research Centre, Newcastle University Centre for Cancer, Translational and Clinical Research Institute, Newcastle upon Tyne, United Kingdom; b Neuro-oncology Section, Division of Hematology/Oncology, Hospital for Sick Children, Toronto, Ontario, Canada; c Department of Applied Sciences, Northumbria University, Newcastle upon Tyne, United Kingdom; d Great North Children's Hospital, Newcastle-upon-Tyne Hospitals NHS Foundation Trust, Newcastle upon Tyne, United Kingdom

**Keywords:** Paediatric oncology, Surgical resection, Prognosis, Molecular groups

## Abstract

**Background:**

Medulloblastoma patients with a sub-total surgical resection (STR; >1.5 cm^2^ primary tumour residuum post-surgery) typically receive intensified treatment. However, the association of STR with poor outcomes has not been observed consistently, questioning the validity of STR as a high-risk disease feature.

**Methods:**

We collected extent of resection (EOR) data from 1110 patients (from UK CCLG centres (n = 416, collected between September 1990 and July 2014) and published (n = 694) cohorts), the largest cohort of molecularly and clinically annotated tumours assembled to specifically assess the significance of EOR. We performed association and univariable/multivariable survival analyses, assessing overall survival (OS) cohort-wide and with reference to the four consensus medulloblastoma molecular groups and clinical features.

**Findings:**

STR was reported in 20% (226/1110) of patients. Non-WNT (p = 0.047), children <5 years at diagnosis (p = 0.021) and metastatic patients (p < 0.0001) were significantly more likely to have a STR. In cohort-wide analysis, STR was associated with worse survival in univariable analysis (p < 0.0001). Examination of specific disease contexts showed that STR was prognostic in univariate analysis for patients receiving cranio-spinal irradiation (CSI) and chemotherapy (p = 0.016) and for patients with Group 3 tumours receiving CSI (p = 0.039). STR was not independently prognostic in multivariable analyses; outcomes for patients who have STR as their only risk-feature are as per standard-risk disease. Specifically, STR was not prognostic in non-metastatic patients that received upfront CSI.

**Interpretation:**

In a cohort of 1100 molecularly characterised medulloblastoma patients, STR (n = 226) predicted significantly lower OS in univariable analysis, but was not an independent prognostic factor. Our data suggest that maximal *safe* resection can continue to be carried out for patients with medulloblastoma and suggest STR should not inform patient management when observed as a sole, isolated risk-feature.

**Funding:**

10.13039/501100000289Cancer Research UK, Newcastle Hospitals Charity, Children’s Cancer North, British Division of the International Academy of Pathology.

**Research in context**

**Evidence before this study**

The current definition of sub-total resection (STR; more than 1.5 cm^2^ tumour volume on post-operative imaging) was defined in the 1980s but persists to this day, and STR remains a commonly adopted prognostic feature for high-risk disease. However, the prognostic significance of STR is controversial as roughly equal numbers of studies identify, or fail to identify, an association between the extent of resection (EOR) and overall survival. The vast majority of these studies did not account for medulloblastoma molecular substructure, now a cornerstone of disease understanding and contemporary diagnostics; interrogating EOR in contemporary molecularly-defined cohorts is therefore urgently required to support an evidence-led clinical strategy.

**Added value of this study**

We assembled and comprehensively analysed a cohort of 1100 medulloblastoma patients (STR, n = 226) to assess the association of EOR with clinico-demographic features both cohort-wide, and in reference to specific disease contexts (demographic, clinical and molecular). Younger patients (<5 years old at diagnosis), and those presenting with metastatic disease, were less likely to achieve a gross total resection. Using this large cohort, we validated the association between EOR and overall survival in univariable analysis, but this was not sustained as an independent risk-factor in multivariable analyses of our UK cohort, in contrast to the consistent behaviour of other, established, high-risk features (i.e. metastatic disease, large-cell/anaplasia (LCA)). Specifically, STR was not prognostic in non-metastatic patients receiving CSI.

**Implications of all the available evidence**

We report one of the largest cohorts of molecularly and clinically annotated patients in this rare tumour type, and use it to address the prognostic significance of EOR. In univariable analysis, STR may hold prognostic relevance in certain specific disease sub-contexts. However, our data does not support its independent prognostic significance when considered alongside establish clinico-molecular high-risk features. These findings provide support for the exclusion of STR as a risk-factor for high-risk medulloblastoma protocols, and therapeutic de-intensification in patients where STR is the sole isolated risk-feature, together aimed at minimising therapy-associated late-effects.

## Introduction

Medulloblastoma is the most common malignant paediatric brain tumour and accounts for around 10% of all cancer deaths in childhood.^1^ Molecular profiling has identified four consensus molecular groups (WNT, SHH, Group 3 and Group 4) and further novel subgroups within the SHH, Group 3 and Group 4 molecular groups.^2^, ^3^, ^4^ Contemporary multimodal treatment for medulloblastoma includes urgent neurosurgical resection for all patients. Current surgical practice seeks to achieve a gross total resection (GTR); where this is not achieved, i.e. >1.5 cm^2^ of primary tumour residuum on post-operative imaging, patients are defined as sub-totally resected (STR).^5^ Dose and regimen of subsequent radiotherapy and adjuvant chemotherapy is stratified according to age and clinical risk; patients with established high-risk disease features (metastatic disease, large cell/anaplastic histology (LCA), *MYC* amplification, *MYCN* amplification and/or *TP53* mutation in SHH tumours) receive intensified therapies.^6^

The definition of 1.5 cm^2^ tumour residuum as the threshold that determines clinically significant sub-total resection was established in the late 1980s, initially based on low-resolution CT imaging.^7^^,^^8^ Gold-standard imaging modalities changed through the 1990s to encompass pre- and post-operative Magnetic Resonance (MR) imaging, which has become uniformly adopted. MR imaging-based studies further validated this same volumetric threshold for sub-total resection, which persists to the current day.^9^^,^^10^ Thompson et al. showed that there was no additional benefit to be gained by distinguishing GTR (no residual tumour) from near total resection (NTR) (<1.5 cm^2^ tumour remaining); GTR and NTR were equivalent in predicting survival.^11^

In their 2018 review of the prognostic value of extent of resection (EOR), Thompson et al. published a systematic review of 50 studies; 16 articles (comprising n = 1489 patients) supported the assignment of STR as a high-risk feature, 20 articles (n = 2335) showed no statistical association between outcomes and STR and 14 articles (n = 2950) which showed mixed results.^12^ However, the vast majority of these studies did not factor medulloblastoma molecular sub-classification^4^ into their assessments, despite these now forming the basis of contemporary medulloblastoma sub-classification and risk-stratification.^4^^,^^13^ The 3/50 articles that did account for molecular group showed inconsistent association of STR with progression free survival (PFS) but no significant relationships with overall survival (OS).^11^^,^^14^^,^^15^ More recent studies have only added to the uncertainty regarding the clinical importance of EOR.^14^^,^^16^, ^17^, ^18^, ^19^ Whilst these have considered the molecular heterogeneity of medulloblastoma, refined surgical practices have driven lower frequencies of STR, under powering the statistical analyses embedded in these studies.^17^^,^^19^ Power calculations performed by Thompson et al. suggested that a 3-year clinical trial to specifically address the clinical significance of STR would require >6000 individual patients.^11^ Given the incidence of medulloblastoma (approximately 1 case per 1 million individuals per year),^5^^,^^6^^,^^20^ and the trend towards lower rates of STR over time, recruiting a cohort of this size with full clinico-pathological and molecular data is not realistically possible. Within reported clinical trials of high-risk medulloblastoma, with associated intensified treatment, the overall survival trends for those patients which are non-metastatic with STR are notably higher than metastatic patients although statistical significance could not be reached.^21^^,^^22^

In Europe at present, the non-infant standard-risk SIOP-PNET5-MB (NCT02066220)^23^ and high-risk SIOP-HR-MB (2018-004250-17)^6^ clinical trials seek to improve risk-stratification by integrating established clinical disease features and molecular features, including molecular group, the status of which are collected prior to commencement of radiotherapy and chemotherapy. Given STR's ambiguous status as a prognostic marker, it has not been included as a high-risk feature, when observed in the absence of other high-risk features, in the eligibility criteria for SIOP-HR-MB. This is in contrast to those high-risk features with a stronger evidence base: LCA pathology, *MYC* amplification, metastatic disease, *TP53* mutation and/or *MYCN* amplification in SHH patients.^5^^,^^6^

Understanding the clinical impact of STR remains critical to inform the management of patients with medulloblastoma, particularly regarding its possible role as a high-risk feature. We thus collected data from 1110 patients, representing the largest cohort of molecularly annotated tumours assembled to date to specifically assess the clinical relevance of EOR in medulloblastoma. We assessed the rates and clinico-molecular correlates of STR, and asked whether there are specific disease and treatment contexts, including within the consensus molecular groups, in which STR has particular clinical relevance. This understanding will be critical to inform the consideration of STR as a risk-factor in the design of future risk-adapted trials, and to direct surgical approaches, in the context of contemporary disease sub-classification.

## Methods

### UK CCLG-based cohort

Tumour material was obtained from UK Children's Cancer and Leukaemia (CCLG) institutions and collaborating centres through the CCLG Tissue Bank. Tumour samples were provided by UK CCLG as part of a CCLG-approved biological study (BS-2007-04). All patients had systematic central clinical review, a confirmed histopathological diagnosis of medulloblastoma, were under 16 years of age at diagnosis and underwent surgical resection between September 1990 and July 2014 (henceforth referred to as the UK cohort). Informed consent was obtained for all patients or their legal guardians and human tumour investigations were conducted with approval from Newcastle/North Tyneside Research Ethics Committee (study reference 07/Q0905/71).

Institutional assessment of EOR was performed using surgical notes and verified using postoperative gadolinium-enhanced T1-weighted MRI. Scan analysis preceded molecular grouping in all cases, meaning radiologists were blinded to the tumour group. GTR was defined as less than 1.5 cm^2^ post-operative tumour residuum and STR as more than 1.5 cm^2^. Metastatic status at diagnosis was determined according to Chang's criteria.^24^ Molecular grouping was performed using Infinium methylation 450 K array according to established protocols.^25^

### Whole cohort

A total cohort of 1110 patients was assembled by combining our UK cohort (n = 416) with available meta-data (including molecular group, age at diagnosis, overall survival, resection and metastatic status) from the MAGIC cohort published by Thompson et al. (n = 694).^11^ The MAGIC cohort consisted of 787 patients who had undergone surgical resection between April 1997 and September 2013. We removed all cases aged 16 and over at diagnosis to harmonise the two collections and create a purely paediatric cohort, termed the ‘Whole cohort’. Thompson et al. classified extent of resection as GTR (no residual tumour), near total resection (NTR) (<1.5 cm^2^ tumour remaining), or STR (≥1.5 cm^2^ tumour remaining); given the equivalence of GTR and NTR in predicting survival, and the absence of this distinction in our cohort, GTR and NTR were combined and classified as GTR. Histopathological annotation and molecular risk-factor (*MYC* amplification, *MYCN* amplification and *TP53* mutation) status were assigned as previously described and were not available for the MAGIC cohort.^26^ Dose of CSI received at diagnosis was categorised into standard dose (<30 Gy) and high dose (≥30 Gy). Similarly, chemotherapy regimens were categorised into high or low dose; high dose was defined as patients in receipt of intensified regimens of sufficient dosage to require stem cell support. Biological sex was collected according to patient self-report.

### Statistics

The association between EOR and clinico-molecular and demographic variables was assessed by performing either Chi-squared tests for categorical variables, or, where the expected frequency for any level of the factor under test was <5, Fisher's exact tests. Comparison of the median survival between cohorts was performed by Wilcoxon–Mann–Whitney test. Overall survival was defined as the interval between diagnosis and death. Log-rank tests were performed with associated Kaplan–Meier plots to compare the impact of extent of resection on overall survival. The assumptions for each statistical analysis were confirmed.

Univariable Cox proportional hazards models were used to investigate the prognostic significance of STR with respect to overall survival across the whole cohort and within specific disease sub-contexts. In accordance with current treatment protocols, patients were categorised by receipt of CSI at diagnosis. Multivariable analysis was used to assess the prognostic significance of STR against other established high-risk MB disease features (metastatic disease, LCA pathology, *MYC*/(*N*) amplification). *TP53* mutation status was not included as a variable in multivariable analysis due to the degree of missing data. Hazard ratios, 95% confidence intervals and significance for overall survival are reported and represented by forest plots.

Statistical significance was taken as p < 0.05. The incidence frequency threshold for statistical testing was 5% for all levels of the factor. Survival analysis was performed using the R package “survival” v3.4. Proportionality of hazards was assessed in Cox models using the ‘cox.zph’ function in the R package “survival” v3.4. All variables tested were proportional with one exception; *MYC* amplification showed non-proportionality of hazards within the non-CSI cohort and consequently multivariable Cox models for this cohort were stratified by *MYC* amplification status using the ‘strata’ function in the R package “survival”. Missing data were assessed using the “mcar_test” function from the R package “naniar” v1.0.0 and confirmed to be ‘missing completely at random’ and omitted from analysis. A sensitivity analysis was performed to evaluate the impact of missing data on survival analysis. Imputation was used to generate a complete dataset using the R package ‘MICE’ v3.16.0 using 10 rounds of predictive means matching for the following variables; Metastatic disease, large-cell/anaplastic histology and *MYC*/(*N*) amplification. The ‘pool’ function from the R package ‘MICE’ v3.16.0 was used to combine and summarise multiply imputed Cox models.

Analyses were performed in R statistical environment (version 4.2.2). Percentages are calculated from samples with available data within the cohort.

### Role of funding source

The funders had no role in study design, data collection, data analysis, data interpretation, writing of the report or the decision of where to publish.

## Results

Our patient cohort (n = 1110) displayed the expected distribution of molecular groups (WNT, 9%; SHH, 27%; Group 3, 24% and Group 4, 40%, Table 1). Median follow up was 5.00 years (0.0–20.91 years). Overall, there was broad equivalence in the incidence of clinico-molecular and demographic features in both contributing cohorts (UK, MAGIC), supporting their combination into a single cohort for analysis (‘Whole cohort’, Table 1). The UK cohort contributed more patients under the age of 5 years at diagnosis (39% vs 33%, p = 0.047), patients treated with focal radiotherapy (12% vs 7%, p = 0.013) and a higher proportion of patients treated with high dose CSI (62% vs 46%, p < 0.0001). The MAGIC cohort contributed more patients with metastatic disease (33% vs 28%, p = 0.046).

**Table 1:** Cohort demographics and clinico-molecular features.

Demographic	Whole cohort	UK cohort	MAGIC cohort	p-value
	n = 1110	n = 416	n = 694	(UK vs MAGIC cohort)
	n (%)	n (%)	n (%)	
**Biological sex**				
Male (M)	717 (65)	268 (64)	449 (65)	0.75
Female (F)	386 (35)	148 (36)	238 (35)	
M:F ratio	1.86:1	1.81:1	1.89:1	
**Age at diagnosis (years)**				
Median [range]	6.78 [0.01–15.97]	6.33 [0.01–15.97]	7.00 [0.33–15.9]	
Under 5	394 (35)	163 (39)	231 (33)	**0.047**
Over 5	716 (65)	253 (61)	463 (67)	
**Molecular group**				
WNT	96 (9)	27 (8)	69 (10)	0.48
SHH	278 (27)	88 (26)	190 (27)	
Group 3	245 (24)	89 (26)	156 (23)	
Group 4	417 (40)	139 (41)	278 (40)	
**Metastatic status at diagnosis**				
M+	332 (31)	114 (28)	218 (33)	**0.046**
M -	736 (69)	300 (72)	436 (67)	
**Extent of resection**				
Sub-total resection (STR)	226 (20)	86 (21)	140 (20)	0.84
Gross total resection (GTR)	884 (80)	330 (79)	554 (80)	
**Receipt of radiotherapy at diagnosis**				
Yes	881 (84)	349 (84)	532 (84)	0.78
No	169 (16)	65 (16)	104 (16)	
**Type of radiotherapy at diagnosis**				
Focal	91 (9)	49 (12)	42 (7)	**0.013**
CSI	790 (75)	300 (72)	490 (77)	
No RTX	169 (16)	65 (16)	104 (16)	
**Dose of CSI at diagnosis**				
Standard (<30Gy)	380 (48)	113 (38)	267 (54)	**<0.0001**
High (≥30Gy)	410 (52)	187 (62)	223 (46)	
**Receipt of radiotherapy and chemotherapy at diagnosis**				
Yes	834 (81)	340 (83)	494 (79)	0.20
No	201 (19)	72 (17)	129 (21)	
**Follow up (years)**				
Median [range]	5.00 [0–20.91]	4.86 [0–19.52]	5.22 [0–20.91]	0.093
**Overall survival (%)**				
5 year OS	67.60%	65.10%	68.99%	0.35
10 year OS	57.80%	56.54%	58.57%	

Data are n (%), median [range]. Chi-squared test, Wilcoxon–Mann–Whitney test and log rank tests were used to compare our UK cohort and the MAGIC cohort. Percentages are calculated from samples with available data within the cohort. Data was assumed to be missing at random and omitted from analysis. p-values reaching statistical significance (p < 0.05) are shown in bold. M+ = Chang's metastatic stage M2 or above; M− = Chang's metastatic stage M0 or M1, OS = overall survival, 95% CI = 95% confidence interval, RTX = radiotherapy.

**Fig. 1:**
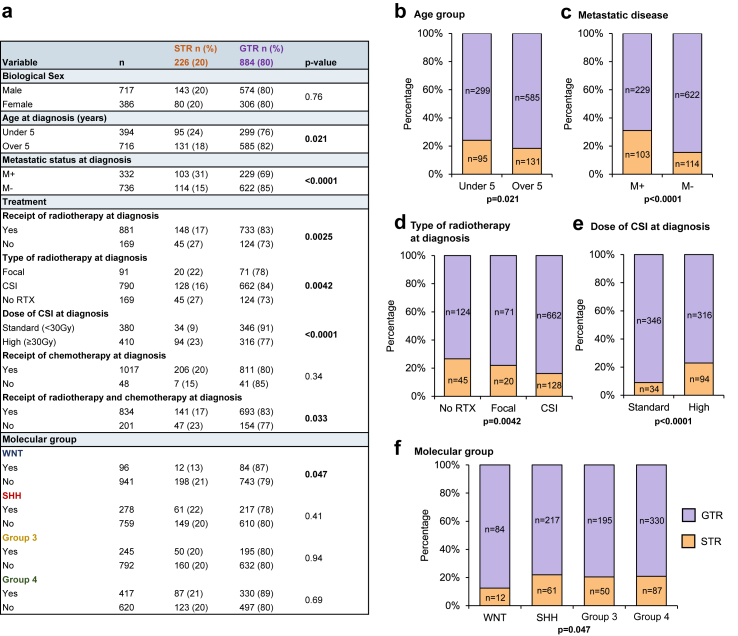
**Association of STR with clinico-pathological and molecular variables. a;** Table showing rates of sub-total resection (STR) in specific demographic and clinico-molecular disease contexts. Data are n (%). Chi-squared test was used to compare the rates of STR between the variables. p-values reaching statistical significance (p < 0.05) are shown in bold. STR = sub-total resection; GTR = gross total resection. M+ = Chang's metastatic stage M2 or above; M− = Chang's metastatic stage M0 or M1; WNT = wnt/wingless; SHH = sonic hedgehog; RTX = radiotherapy **b–f;** Stacked bar charts representing the significant association observed between STR and the following disease contexts: patients under 5 years of age at diagnosis, patients presenting with metastatic disease at diagnosis, type of radiotherapy received at diagnosis, dose of CSI received at diagnosis and molecular group. Bar label = count (n).

The rate of STR across the whole cohort was 20% (226/1110). We assessed the association between rates of STR and specific demographic and disease contexts (sex, age at diagnosis, metastatic disease, treatment and molecular group; Fig. 1a). Patients who were (1) under 5 years at diagnosis (p = 0.021), (2) had metastatic disease (p < 0.0001), (3) did not receive radiotherapy (p = 0.0025) or (4) received high dose CSI (p < 0.0001) were associated with higher rates of STR (Fig. 1b–e). The WNT group, which carries a favourable disease risk,^27^ had lower rates of STR in comparison to the other groups (p = 0.047) (Fig. 1f). These findings were broadly recapitulated in the UK cohort when assessed in isolation (Supplementary Figure S1).

**Fig. 2:**
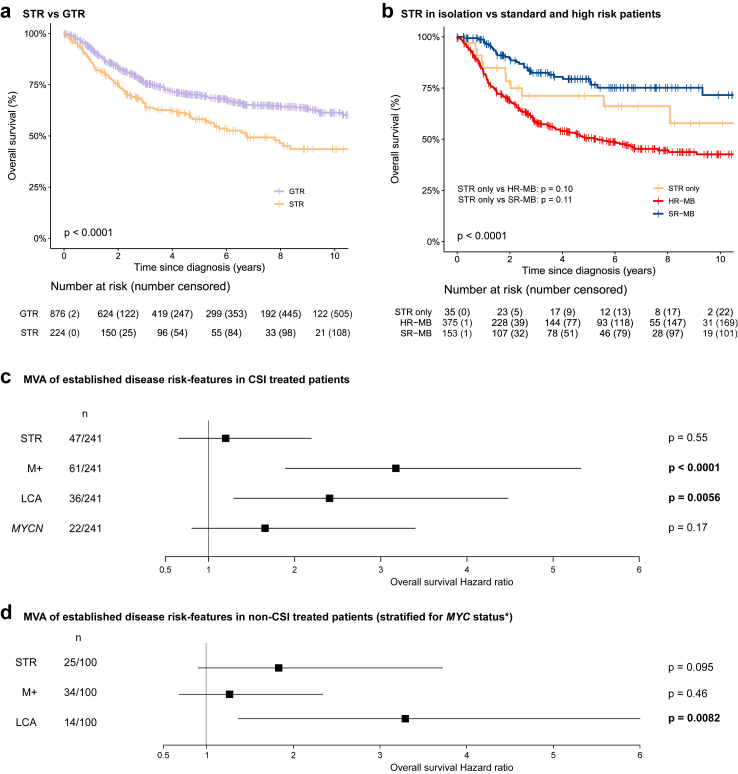
**STR is associated with overall survival in the whole cohort and the UK cohort. a;** Kaplan–Meier estimates of overall survival in the whole cohort based on EOR, p-value = log-rank test. **b;** Kaplan–Meier estimates of overall survival in the whole cohort comparing patients with STR as their only high-risk feature (STR only) to patients with other high-risk features (HR-MB; presence of any of LCA pathology, metastatic disease, *MYC*/*(N)* amplification (excluding *MYCN* amplification in Group 4), *TP53* mutant SHH-disease) or none (SR-MB; only those patients with full annotation for these features), p-value = log-rank test. At-risk tables are shown in two-year increments with number of patients censored in parentheses. **c and d;** Forest plots of multivariable (MVA) Cox regression hazard ratios, 95% confidence intervals and p-values of established high-risk disease features in UK patients receiving CSI **(c)** and UK patients not receiving CSI **(d)**, significant features are highlighted in bold. ∗The Cox model is stratified by *MYC* amplification status for the non-CSI multivariable Cox regression due to its non-proportionality of hazards.

We next investigated the prognostic significance of EOR in the whole cohort (Fig. 2) and in the UK cohort (Supplementary Figure S2). STR was associated with poorer outcomes in the whole cohort (5-year OS, STR (58.12%) vs GTR (70.00%), p < 0.0001; Fig. 2a) and in the UK cohort (5-year OS, STR (53.37%) vs GTR (68.47%), p = 0.021; Supplementary Figure S2a). We then proceeded to assess the prognostic significance of STR when observed as an isolated risk-feature. In patients from the whole cohort where the clinical annotation was available, survival rates of STR-only patients were as per standard-risk disease (5-year OS 71.17% vs 79.40%; p = 0.11, Fig. 2b). Moreover, STR was not independently prognostic at the multivariable level, independent of receipt of CSI (CSI: HR 1.20, 0.66–2.19 [95% CI], p = 0.55, Fig. 2c; CSI-naïve: HR 1.83, 0.90–3.72 [95% CI], p = 0.095; Fig. 2d), and in contrast to other established clinico-pathological factors; metastatic disease (in CSI treated patients: HR 3.18, 1.89–5.32 [95% CI], p < 0.0001) and LCA pathology (in CSI treated: HR 2.41, 1.29–4.48 [95% CI], p = 0.0056; in CSI-naïve patients: HR 3.29, 1.36–7.96 [95% CI], p = 0.0082). Sensitivity analyses confirmed that these findings were not influenced by missing data (Supplementary Tables S4 and S5). In CSI-treated STR patients, CSI dose was not significantly associated with outcome in either the whole cohort (p = 0.35, Supplementary Figure S3a) or the UK cohort (p = 0.087, Supplementary Figure S3c).

**Fig. 3:**
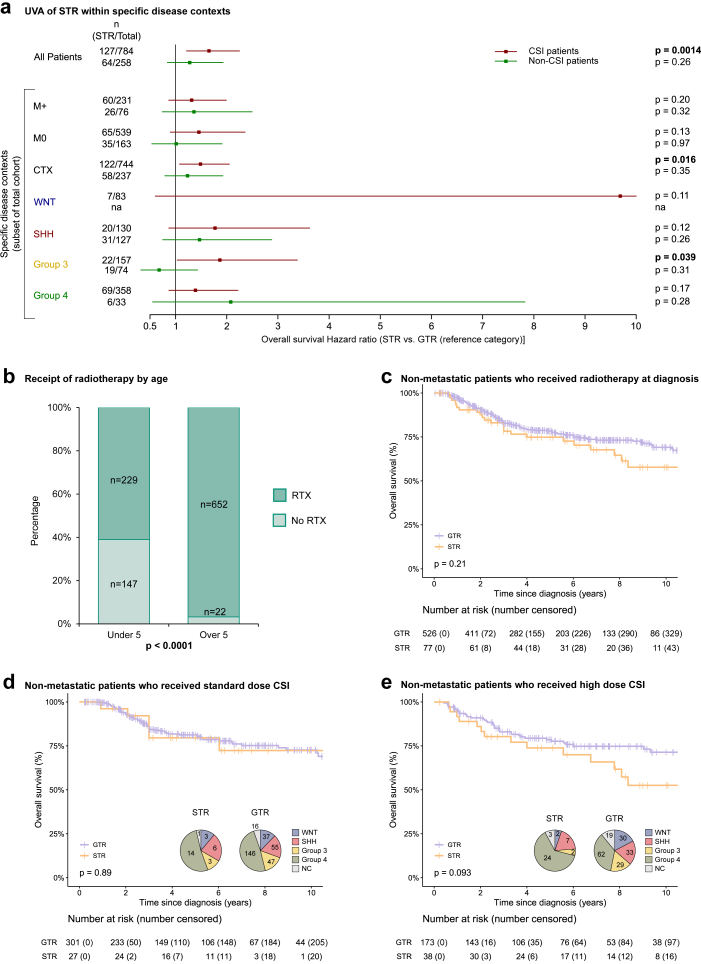
**Prognostic associations of STR vary according to clinico-molecular context. a;** Forest plot of the univariable (UVA) Cox regression hazard ratios, 95% confidence interval and p-values of STR within each disease specific sub-context (reference group = GTR). Red lines represent analyses of patients receiving CSI, green lines represent analyses of patients not receiving CSI. In WNT patients the upper 95% CI was over 10 and not shown on this plot. **b;** Stacked bar chart representing the significant association observed between age and patients who received radiotherapy at diagnosis. Bar label = count (n), p-value is from Chi-squared test. **c;** Kaplan–Meier estimates of overall survival based on EOR in non-metastatic patients who received radiotherapy at diagnosis, p-value = log-rank test. Kaplan–Meier estimates of overall survival based on EOR in non-metastatic patients who received **d;** standard dose or **e;** high dose CSI, p-value = log-rank test. At-risk tables are shown in two-year increments with number of patients censored in parentheses. Pie charts represent subgroup distribution; blue = WNT; red = SHH; yellow = Group 3; green = Group 4; grey = not classified.

To further refine the prognostic significance of STR, we next assessed the associations between STR and OS in specific disease sub-contexts and found that STR was significantly associated with survival in patients that received treatment (CSI and chemotherapy, HR 1.46, 0.90–2.36 [95% CI], p = 0.016) and Group 3 patients that received CSI at diagnosis (HR 1.87, 1.03–3.38 [95% CI], p = 0.039, Fig. 3a), however these findings were not replicated in analysis of the UK cohort (Supplementary Figure S4a). As expected, age and radiotherapy receipt were inter-dependent; patients over the age of 5 years at diagnosis were strongly associated with receiving radiotherapy (p < 0.0001, Fig. 3b), representing conventional treatment paradigms.

We then focused on the impact of STR in non-metastatic patients that were treated with radiotherapy. In this specific disease context, STR was not significantly associated with outcome either in the whole cohort (p = 0.21, Fig. 3c) or within the four molecular groups, including Group 3 patients (Supplementary Figure S5a and d). Importantly, STR behaviour was independent of treatment dose; there was no association with OS in non-metastatic patients who received either standard dose (p = 0.89, Fig. 3d) or high dose (p = 0.093, Fig. 3e) CSI. These findings were recapitulated in the UK cohort (Supplementary Figure S4c–e) and within the molecular groups (Supplementary Figure S6). UK non-metastatic STR patients who received standard-dose CSI had no other high-risk features, with the exception of a single Group 4 patient who had a tumour with LCA pathology (Supplementary Figure S7).

Chemotherapy dose was available for the UK cohort. As expected, patients under 5 years old at diagnosis were enriched for the receipt of high dose chemotherapy (Supplementary Figure S8a). Chemotherapy dose did not associate with OS in STR patients from the UK cohort (p = 0.27, Supplementary Figure S8b).

## Discussion

Over the last 25 years, STR has contributed to the definition of high-risk disease in medulloblastoma. Recently, however, the status of STR as an independent high-risk feature has been questioned. This question is clinically important - if EOR does not contribute significantly to risk-stratification, those patients for whom STR is their only high-risk feature could be spared from intensified treatment regimens and the associated increase in life-limiting deficits in the quality of their survivorship. To address this clinical need, we assembled and assessed a cohort of 1100 medulloblastoma patients with clinical and molecular annotation, including molecular group, to assess any association of STR with established disease-related features, its prognostic utility and whether any relationships observed were dependent upon specific disease contexts.

We saw an increased incidence of STR in patients younger than 5 years at diagnosis (who were less likely to receive radiotherapy or have a WNT tumour) and in patients with metastatic disease. This possibly reflects inherent complexity of paediatric neurosurgery in the youngest patients and where disseminated disease is present. Also, the perception of limited additional benefit from a more aggressive surgical approach in these challenging high-risk disease contexts may have contributed to a more conservative surgical philosophy.

Whilst we did confirm the survival disadvantage conferred by STR in univariable analysis, this significance was not sustained in multivariable analyses when assessed alongside established high-risk features (metastatic disease, LCA pathology), independent of treatment. Specifically, there was no significant association between STR and survival in the context of non-metastatic patients that received radiotherapy, in the whole cohort or within each molecular group. Moreover, survival outcomes for patients whose only risk-feature was STR were as per standard-risk disease. The significant univariable association between STR and survival in Group 3 tumours with receipt of CSI suggested STR may have significance in certain settings, though, further exploratory analysis did not support this. We recognise that, due to the power limitations of the study, we cannot conclusively rule out an association between EOR and survival in all disease contexts. In STR patients with no other high-risk features, the balance of therapeutic morbidity associated with intensified treatment regimens and the perceived modest survival benefit warrants specific review within a multi-disciplinary team setting, if STR continues to be an exclusion criterion within current clinical trials. Regarding the notion of second-look surgery to improve prognosis, our data indicates that in most clinical settings, if decompression has been achieved as a primary intervention, the residuum has a limited impact on survival outcome and has not been shown to be independently prognostic in any of the analysed specific disease contexts. Whether there is an additional benefit to neurological/neurocognitive outcomes following less aggressive surgery remains to be assessed in future clinical trials.

Limitations of our study include its retrospective nature and discrepancies in available variables between our UK cohort and the MAGIC cohort, meaning some analyses could only be performed in our well-annotated UK cohort. There is also the ongoing challenge that, despite being the largest cohort to our knowledge to assess EOR in medulloblastoma, the small effect size and the low rate of STR together confer a lack of power to analyse the specific disease contexts of interest. Where we found no prognostic association with STR, we recognise the likelihood that the majority of STR patients will have been treated in accordance with higher intensity regimens, an important consideration when reviewing opportunities for therapy de-escalation. Reassuringly, where data was available, outcomes for those STR patients that received lower-intensity treatment were equivalent to those patients receiving high-dose regimens. Given the incidence of medulloblastoma and the low and improving rates of STR with advancements in neurosurgical practices, assembling a cohort to validate this finding would not be feasible.

**Fig. 4:**
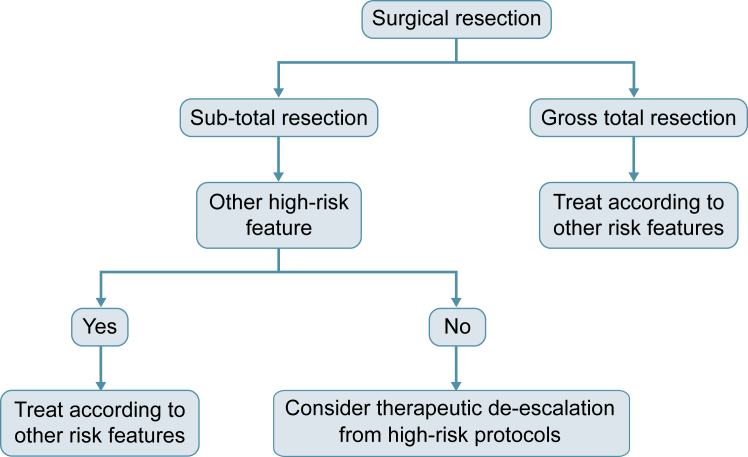
**Proposed management of medulloblastoma patients where STR is the sole high-risk feature**.

In conclusion, findings from our study support the view that the primary goal of neurosurgical resection should continue to be maximal *safe* resection. The lack of independent prognostic significance supports the exclusion of STR as a stand-alone risk-factor for high-risk treatment protocols and suggests treatment according to the presence of any of the other identified high-risk features. These findings provide practical utility in the handling of those patients with STR ineligible for current European clinical trials and we offer a framework to guide the clinical management of STR (Fig. 4).

## Contributors

SB, DH and SCC designed the study. SD, CK, JG, ECS, SB, DH and SCC performed the analysis. CK, JG, DH and SCC wrote the manuscript. SD, CK, JG, ECS SB, VR, DH and SCC reviewed draft versions of the manuscript. All authors contributed to and approved the final manuscript and all authors had full access to the data; CK, JG, ECS and DH accessed and verified underlying data reported in the manuscript.

## Data sharing statement

Subsets of this data were part of a previous study.^11^ All remaining data can be shared after approval of the corresponding author, following a reasonable submitted request.

## Declaration of interests

We declare no competing interests.
