# Supplementary material for: The clinical significance of sub-total surgical resection in childhood medulloblastoma: a multi-cohort analysis of 1100 patients
Source: eClinicalMedicine. 2024 Feb 14;69:102469. doi: 10.1016/j.eclinm.2024.102469 (PMC10875250; doi:10.1016/j.eclinm.2024.102469)
Supplement: Supplementary Material [file mmc1.pdf]

*The clinical significance of sub-total surgical resection in childhood medulloblastoma: a multi-cohort analysis of 1100 patients*

*Keeling et al.*

*Supplementary material*

**Supplementary Figure 1**

**a**

| Variable | n | STR n (%) 68 (17) | GTR n (%) 330 (83) | p-value |
|---|---|---|---|---|
| **Gender** | | | | |
| Male | 268 | 54 (20) | 214 (80) | 0.72 |
| Female | 148 | 32 (22) | 116 (78) | |
| **Age at diagnosis (years)** | | | | |
| Under 5 | 163 | 39 (24) | 124 (76) | 0.19 |
| Over 5 | 253 | 47 (19) | 206 (81) | |
| **High-risk features** | | | | |
| **Metastatic disease at diagnosis** | | | | |
| M+ | 114 | 37 (32) | 77 (68) | **0.00030** |
| M- | 300 | 49 (16) | 251 (84) | |
| **LCA pathology** | | | | |
| Yes | 52 | 9 (17) | 43 (83) | 0.51 |
| No | 314 | 67 (21) | 247 (79) | |
| ***MYC* amplification** | | | | |
| Yes | 20 | 3 (15) | 17 (85) | 0.78 |
| No | 362 | 79 (21) | 289 (79) | |
| ***MYCN* amplification** | | | | |
| Yes | 25 | 3 (12) | 22 (88) | 0.32 |
| No | 362 | 79 (22) | 283 (78) | |
| ***TP53* mutation** | | | | |
| Yes | 19 | 1 (5) | 18 (95) | 0.13 |
| No | 188 | 38 (20) | 150 (80) | |
| **Treatment** | | | | |
| **Receipt of radiotherapy at diagnosis** | | | | |
| Yes | 349 | 67 (19) | 282 (81) | 0.067 |
| No | 65 | 19 (29) | 46 (71) | |
| **Type of radiotherapy at diagnosis** | | | | |
| Focal | 49 | 11 (22) | 38 (88) | |
| CSI | 300 | 56 (19) | 244 (81) | 0.16 |
| No RTX | 49 | 11 (22) | 38 (78) | |
| **Dose of CSI at diagnosis** | | | | |
| Standard (<30Gy) | 113 | 9 (8) | 104 (92) | **0.00022** |
| High (≥30Gy) | 187 | 47 (25) | 140 (75) | |
| **Receipt of chemotherapy at diagnosis** | | | | |
| Yes | 401 | 81 (19) | 320 (81) | 0.68 |
| No | 12 | 3 (25) | 9 (75) | |
| **Dose of chemotherapy** | | | | |
| Standard | 310 | 53 (17) | 257 (83) | 0.034 |
| High | 63 | 18 (29) | 45 (71) | |
| **Receipt of radiotherapy and chemotherapy at diagnosis** | | | | |
| Yes | 340 | 64 (19) | 276 (81) | 0.087 |
| No | 72 | 20 (28) | 52 (72) | |
| **Group** | | | | |
| **WNT** | | | | |
| Yes | 27 | 1 (4) | 26 (96) | na |
| No | 316 | 69 (22) | 247 (78) | |
| **SHH** | | | | |
| Yes | 88 | 16 (18) | 72 (82) | 0.55 |
| No | 255 | 54 (21) | 201 (79) | |
| **Group 3** | | | | |
| Yes | 89 | 20 (22) | 69 (78) | 0.57 |
| No | 254 | 50 (20) | 204 (80) | |
| **Group 4** | | | | |
| Yes | 139 | 33 (24) | 106 (76) | 0.21 |
| No | 204 | 37 (18) | 167 (82) | |

**b Metastatic status**

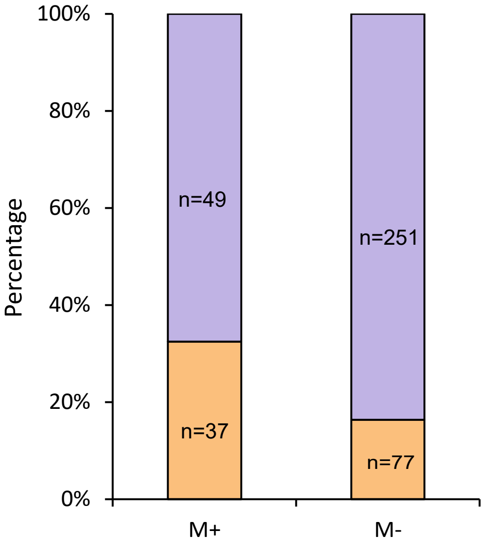


**p = 0.00030**

**c Dose of CSI and diagnosis**

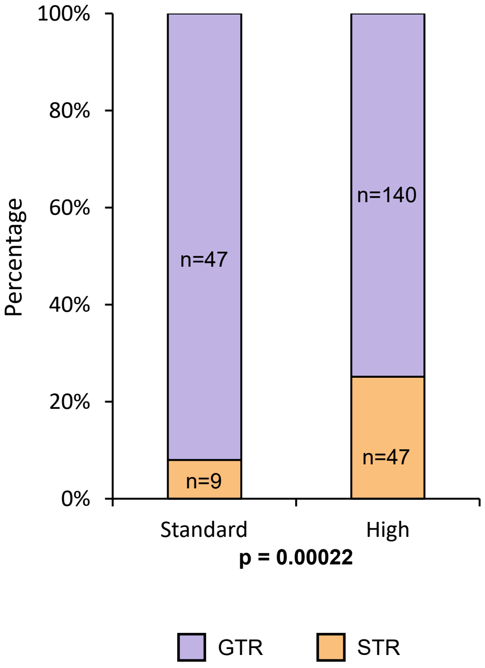


**p = 0.00022**

## Supplementary Figure 2

### a STR vs GTR (UK cohort)

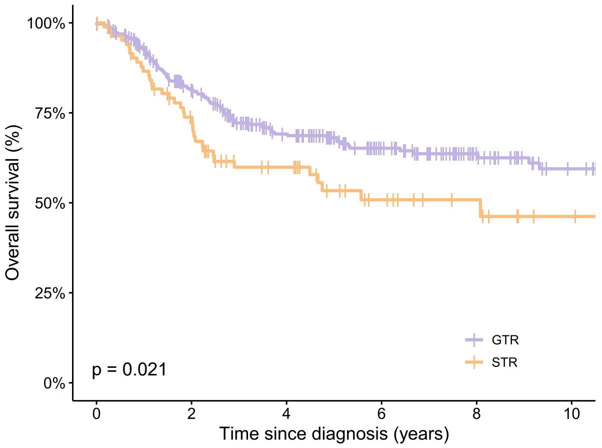


Number at risk (number censored)

| | | | | | | |
|---|---|---|---|---|---|---|
| GTR | 329 (1) | 222 (52) | 152 (90) | 93 (142) | 56 (177) | 34 (196) |
| STR | 85 (0) | 55 (9) | 35 (19) | 18 (32) | 11 (39) | 4 (45) |

### b STR in isolation vs standard and high risk patients (UK cohort)

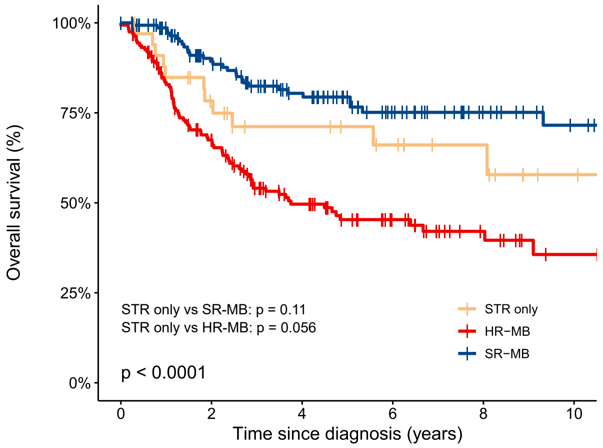


Number at risk (number censored)

| | | | | | | |
|---|---|---|---|---|---|---|
| STR only | 35 (0) | 23 (5) | 17 (9) | 12 (13) | 8 (17) | 2 (22) |
| HR−MB | 162 (0) | 95 (18) | 55 (34) | 31 (54) | 17 (66) | 8 (73) |
| SR−MB | 153 (1) | 107 (32) | 78 (51) | 46 (79) | 28 (97) | 19 (105) |

## Supplementary Figure 3

### a CSI treated STR patients

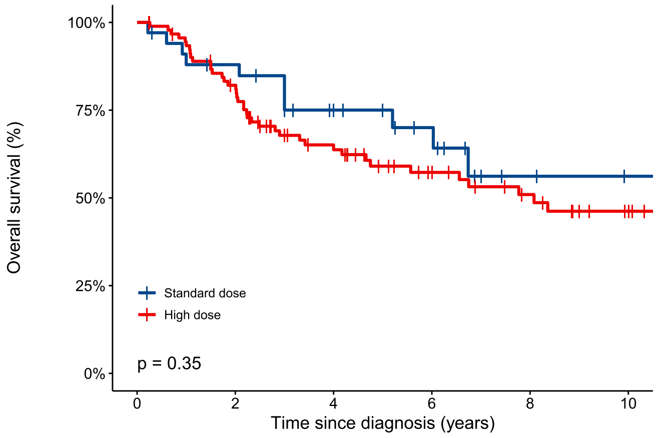


Number at risk (number censored)

| | 0 | 2 | 4 | 6 | 8 | 10 |
|---|---|---|---|---|---|---|
| Standard dose | 34 (0) | 28 (2) | 20 (8) | 12 (13) | 3 (20) | 1 (22) |
| High dose | 93 (0) | 71 (6) | 47 (16) | 30 (29) | 22 (33) | 13 (40) |

### b CSI treated GTR patients

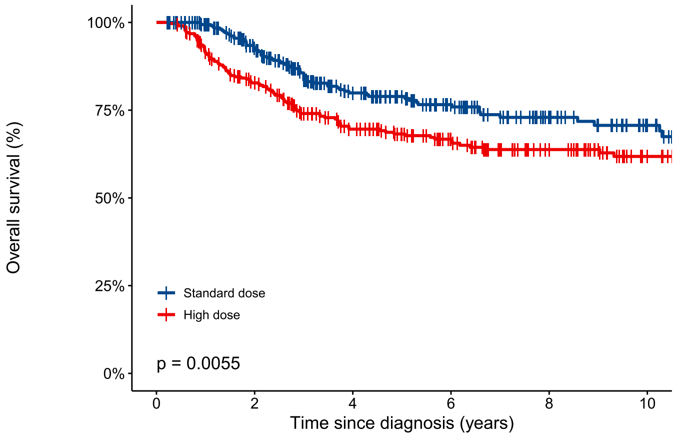


Number at risk (number censored)

| | 0 | 2 | 4 | 6 | 8 | 10 |
|---|---|---|---|---|---|---|
| Standard dose | 344 (0) | 264 (58) | 165 (126) | 118 (167) | 74 (208) | 48 (231) |
| High dose | 313 (0) | 238 (25) | 166 (60) | 121 (104) | 81 (136) | 53 (162) |

### c CSI treated STR patients (UK cohort)

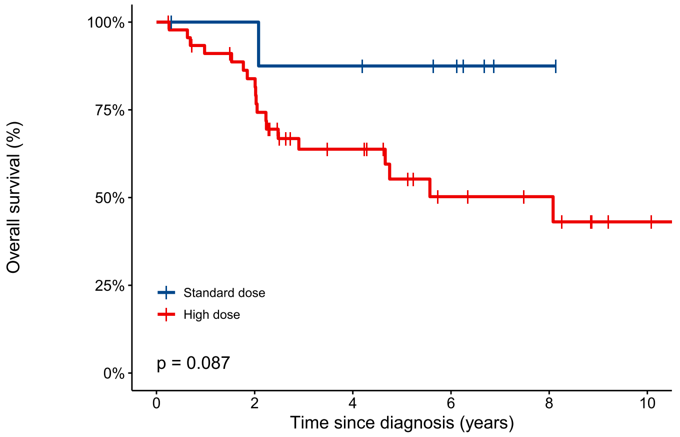


Number at risk (number censored)

| | 0 | 2 | 4 | 6 | 8 | 10 |
|---|---|---|---|---|---|---|
| Standard dose | 9 (0) | 8 (1) | 7 (1) | 5 (3) | 1 (7) | 0 (8) |
| High dose | 46 (0) | 35 (4) | 20 (11) | 9 (19) | 7 (21) | 2 (25) |

### d CSI treated GTR patients (UK cohort)

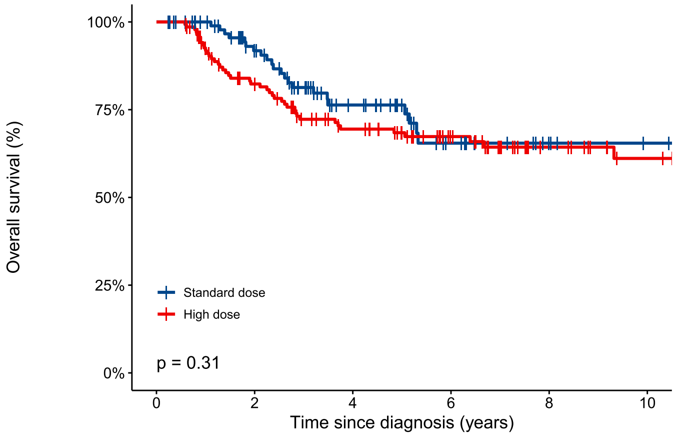


Number at risk (number censored)

| | 0 | 2 | 4 | 6 | 8 | 10 |
|---|---|---|---|---|---|---|
| Standard dose | 104 (0) | 73 (24) | 41 (45) | 20 (62) | 10 (72) | 7 (75) |
| High dose | 140 (0) | 101 (17) | 74 (28) | 48 (52) | 27 (71) | 18 (79) |

**Supplementary Figure 4**

**a UVA of STR within MB disease subsets (UK cohort)**

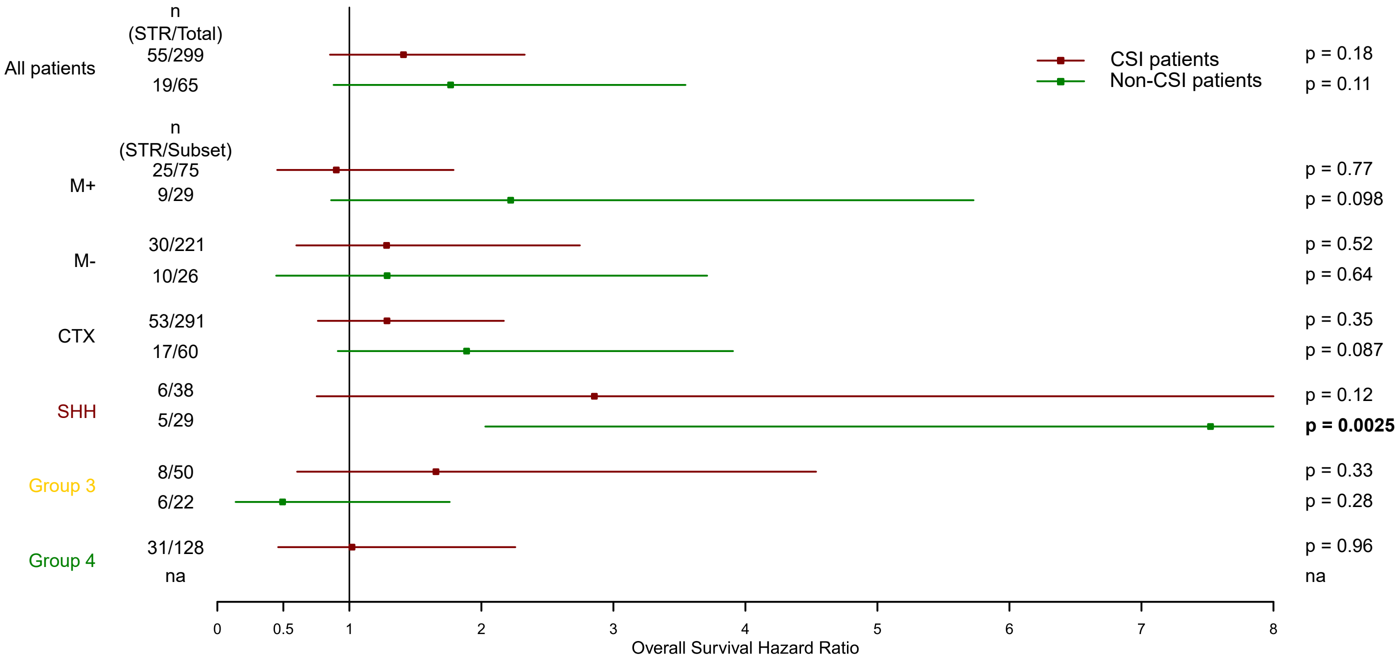


**b Receipt of radiotherapy by age (UK cohort)**

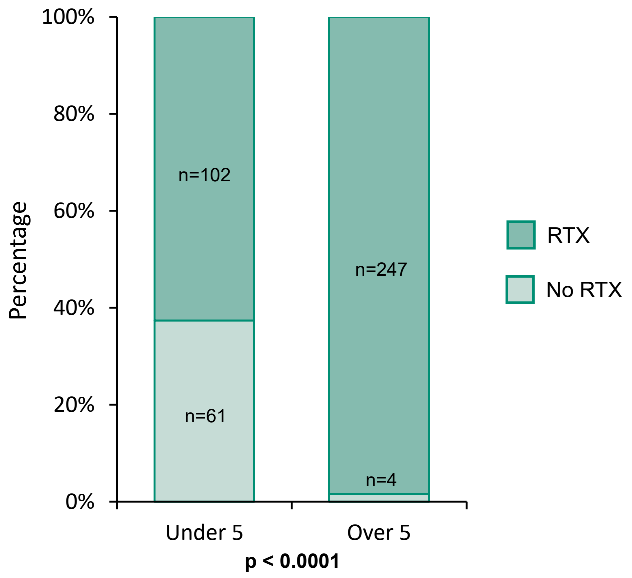


**c Non-metastatic patients who received RTX (UK cohort)**

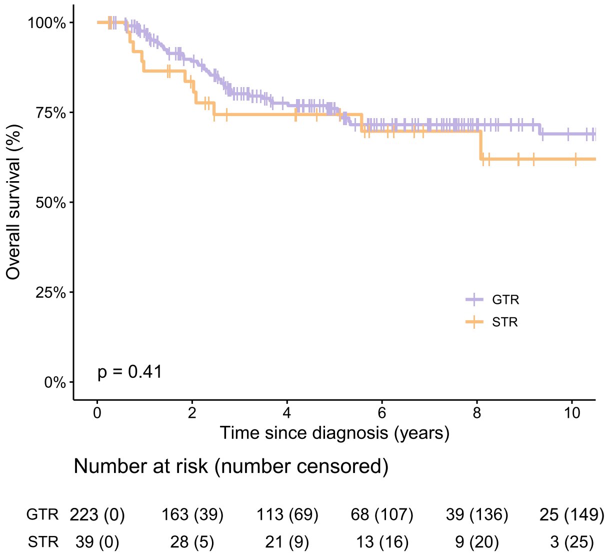


**d Non-metastatic patients who received standard dose CSI (UK cohort)**

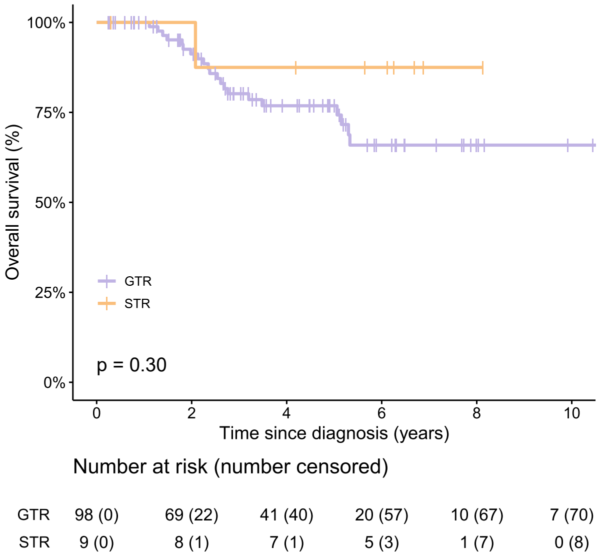


**e Non-metastatic patients who received high dose CSI (UK cohort)**

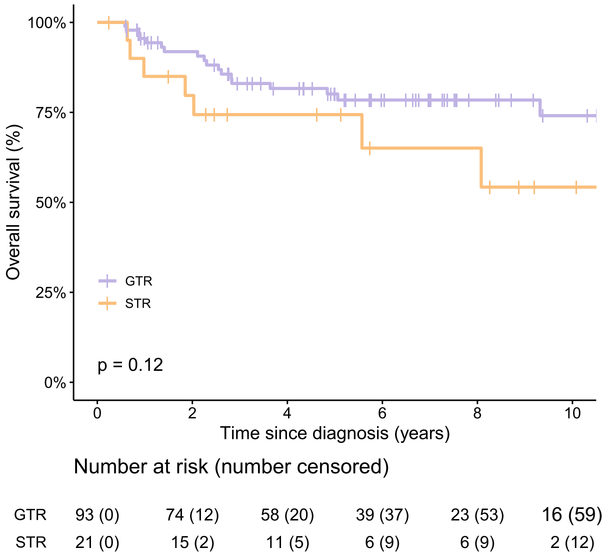

**Supplementary Figure 5**

**a Non-metastatic Group 3 patients who received radiotherapy at diagnosis**

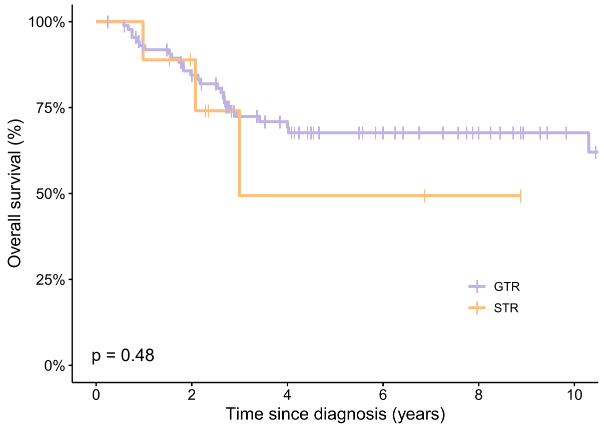


Number at risk (number censored)

| | | | | | | |
|---|---|---|---|---|---|---|
| GTR | 89 (0) | 68 (9) | 44 (22) | 31 (34) | 21 (44) | 12 (52) |
| STR | 9 (0) | 6 (2) | 2 (4) | 2 (4) | 1 (5) | 0 (6) |

**b Non-metastatic Group 4 patients who received radiotherapy at diagnosis**

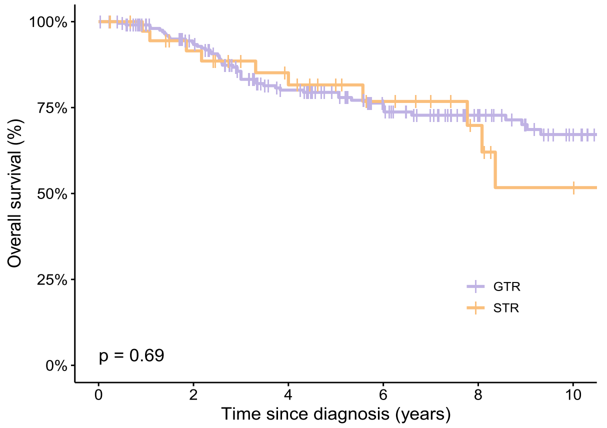


Number at risk (number censored)

| | | | | | | |
|---|---|---|---|---|---|---|
| GTR | 215 (0) | 176 (27) | 123 (56) | 89 (86) | 60 (111) | 39 (129) |
| STR | 39 (0) | 31 (5) | 24 (10) | 15 (17) | 9 (22) | 5 (24) |

**c Non-metastatic WNT patients who received radiotherapy at diagnosis**

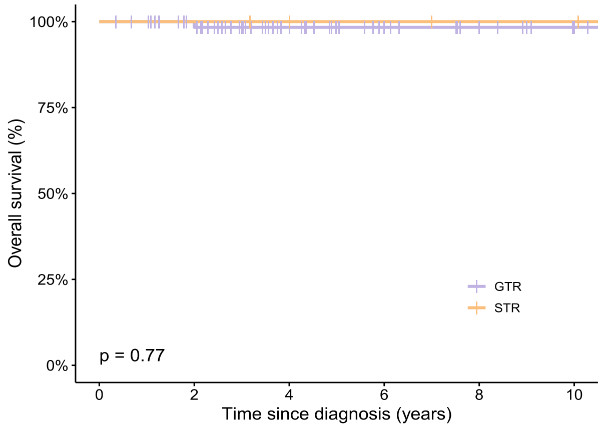


Number at risk (number censored)

| | | | | | | |
|---|---|---|---|---|---|---|
| GTR | 70 (0) | 60 (10) | 36 (34) | 24 (46) | 18 (52) | 12 (58) |
| STR | 5 (0) | 5 (0) | 4 (2) | 3 (2) | 2 (3) | 2 (3) |

**d Non-metastatic SHH patients who received radiotherapy at diagnosis**

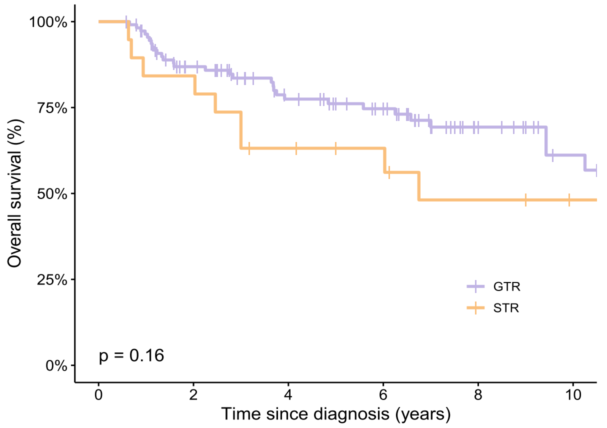


Number at risk (number censored)

| | | | | | | |
|---|---|---|---|---|---|---|
| GTR | 112 (0) | 84 (14) | 62 (28) | 49 (40) | 25 (62) | 16 (69) |
| STR | 19 (0) | 16 (0) | 11 (1) | 9 (3) | 6 (4) | 4 (6) |

## Supplementary Figure 6

**a Non-metastatic Group 3 patients who received radiotherapy at diagnosis - UK cohort only**

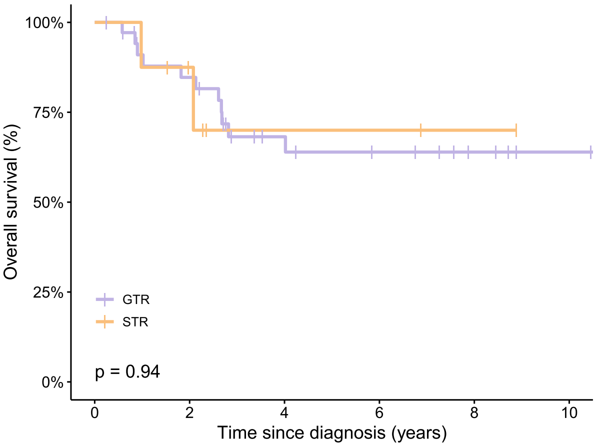


Number at risk (number censored)

| | 0 | 2 | 4 | 6 | 8 | 10 |
|---|---|---|---|---|---|---|
| GTR | 36 (0) | 27 (4) | 16 (10) | 13 (12) | 9 (16) | 6 (19) |
| STR | 8 (0) | 5 (2) | 2 (4) | 2 (4) | 1 (5) | 0 (6) |

**b Non-metastatic Group 4 patients who received radiotherapy at diagnosis - UK cohort only**

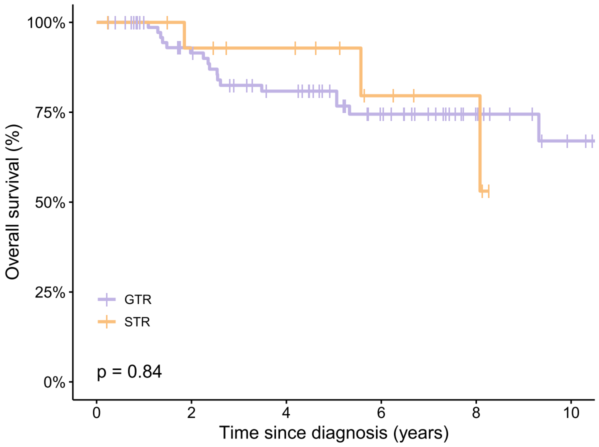


Number at risk (number censored)

| | 0 | 2 | 4 | 6 | 8 | 10 |
|---|---|---|---|---|---|---|
| GTR | 81 (0) | 62 (13) | 49 (19) | 30 (35) | 15 (50) | 7 (57) |
| STR | 16 (0) | 13 (2) | 11 (4) | 5 (9) | 3 (11) | 0 (13) |

**c Non-metastatic SHH patients who received radiotherapy at diagnosis - UK cohort only**

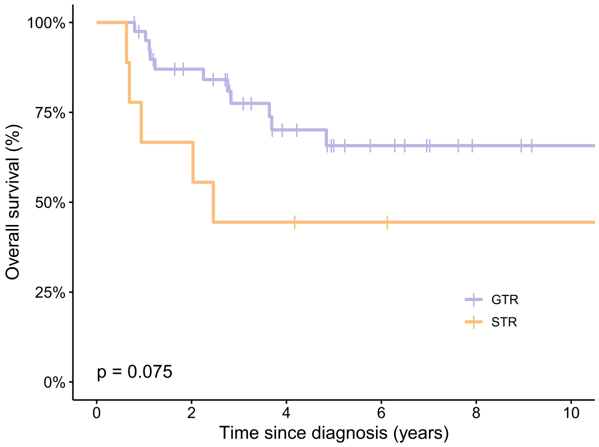


Number at risk (number censored)

| | 0 | 2 | 4 | 6 | 8 | 10 |
|---|---|---|---|---|---|---|
| GTR | 41 (0) | 30 (6) | 17 (14) | 10 (20) | 4 (26) | 2 (28) |
| STR | 9 (0) | 6 (0) | 4 (0) | 3 (1) | 2 (2) | 2 (2) |

**Supplementary Figure 7**

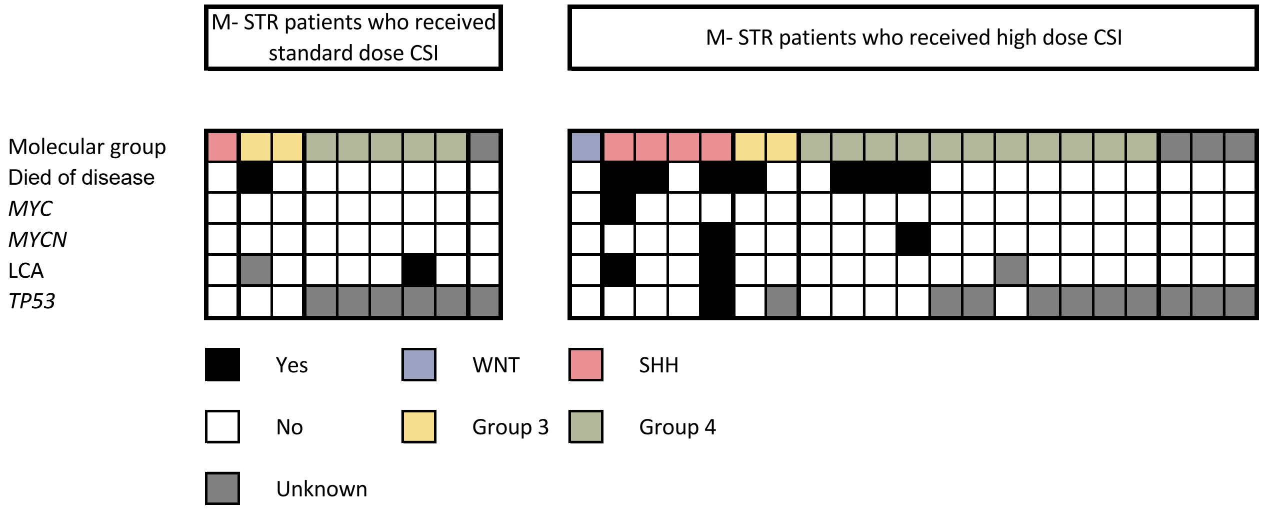

**Supplementary Figure 8**

**a Chemotherapy dose recieved by age (UK cohort)**

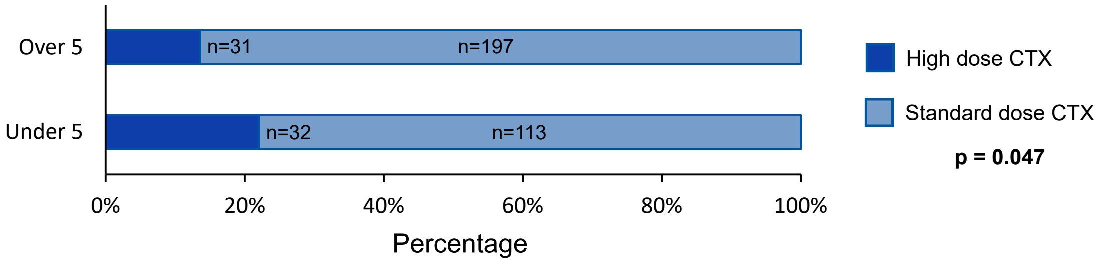


**b STR patients (UK cohort)**

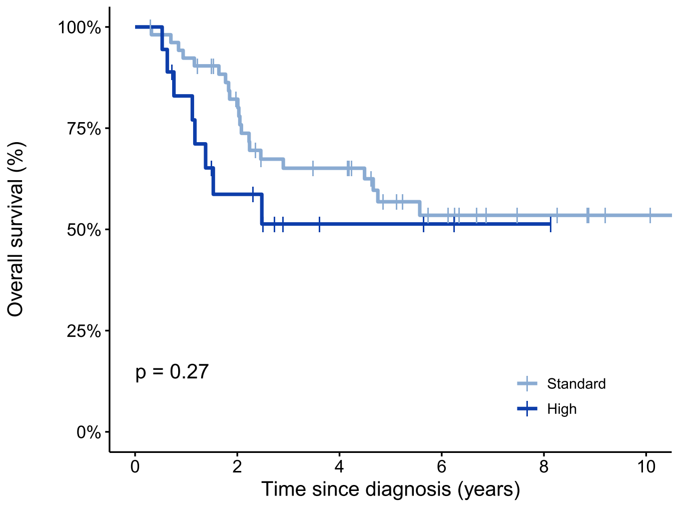


Number at risk (number censored)

| | 0 | 2 | 4 | 6 | 8 | 10 |
|---|---|---|---|---|---|---|
| Standard | 53 (0) | 39 (5) | 28 (8) | 15 (17) | 9 (23) | 4 (28) |
| High | 18 (0) | 9 (2) | 3 (7) | 2 (8) | 1 (9) | 0 (10) |

**c GTR patients (UK cohort)**

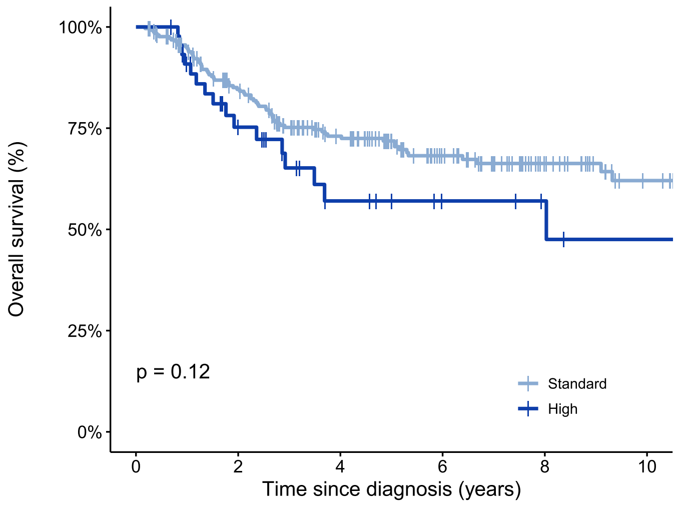


Number at risk (number censored)

| | 0 | 2 | 4 | 6 | 8 | 10 |
|---|---|---|---|---|---|---|
| Standard | 257 (0) | 184 (37) | 132 (65) | 79 (111) | 44 (144) | 25 (161) |
| High | 45 (0) | 25 (10) | 13 (17) | 8 (22) | 6 (24) | 4 (25) |

## Supplementary Table 1

| Variable | n | STR n (%) 68 (17) | GTR n (%) 330 (83) | p-value |
|---|---|---|---|---|
| **Gender** | | | | |
| Male | 268 | 54 (63) | 214 (65) | 0.72 |
| Female | 148 | 32 (37) | 116 (35) | |
| **Age at diagnosis (years)** | | | | |
| Under 5 | 163 | 39 (45) | 124 (38) | 0.19 |
| Over 5 | 253 | 47 (55) | 206 (62) | |
| **High-risk fetures** | | | | |
| **Metastatic disease at diagnosis** | | | | |
| M+ | 114 | 37 (43) | 77 (23) | **0.00030** |
| M- | 300 | 49 (57) | 251 (77) | |
| **LCA pathology** | | | | |
| Yes | 52 | 9 (12) | 43 (15) | 0.51 |
| No | 314 | 67 (88) | 247 (85) | |
| ***MYC* amplification** | | | | |
| Yes | 20 | 3 (4) | 17 (6) | 0.78 |
| No | 362 | 79 (96) | 289 (94) | |
| ***MYCN* amplification** | | | | |
| Yes | 25 | 3 (4) | 22 (7) | 0.32 |
| No | 362 | 79 (96) | 283 (93) | |
| ***TP53* mutation** | | | | |
| Yes | 19 | 1 (3) | 18 (11) | 0.13 |
| No | 188 | 38 (97) | 150 (89) | |
| **Treatment** | | | | |
| **Receipt of radiotherapy at diagnosis** | | | | |
| Yes | 349 | 67 (78) | 282 (86) | 0.067 |
| No | 65 | 19 (22) | 46 (14) | |
| **Type of radiotherapy at diagnosis** | | | | |
| Focal | 49 | 11 (14) | 38 (12) | |
| CSI | 300 | 56 (72) | 244 (76) | 0.16 |
| No RTX | 49 | 11 (14) | 38 (12) | |
| **Dose of CSI at diagnosis** | | | | |
| Standard (<30Gy) | 113 | 9 (16) | 104 (43) | **0.00022** |
| High (≥30Gy) | 187 | 47 (84) | 140 (57) | |
| **Receipt of chemotherapy at diagnosis** | | | | |
| Yes | 401 | 81 (96) | 320 (97) | 0.68 |
| No | 12 | 3 (4) | 9 (3) | |
| **Dose of chemotherapy** | | | | |
| Standard | 310 | 53 (75) | 257 (85) | **0.034** |
| High | 63 | 18 (25) | 45 (15) | |
| **Receipt of radiotherapy and chemotherapy at diagnosis** | | | | |
| Yes | 340 | 64 (76) | 276 (84) | 0.087 |
| No | 72 | 20 (24) | 52 (16) | |
| **Group** | | | | |
| **WNT** | | | | |
| Yes | 27 | 1 (1) | 26 (10) | na |
| No | 316 | 69 (99) | 247 (90) | |
| **SHH** | | | | |
| Yes | 88 | 16 (23) | 72 (26) | 0.55 |
| No | 255 | 54 (77) | 201 (74) | |
| **Group 3** | | | | |
| Yes | 89 | 20 (29) | 69 (25) | 0.57 |
| No | 254 | 50 (71) | 204 (75) | |
| **Group 4** | | | | |
| Yes | 139 | 33 (47) | 106 (39) | 0.21 |
| No | 204 | 37 (53) | 167 (61) | |

**Supplementary Table 2**

| | | CSI treated patients | | | | | |
|---|---|---|---|---|---|---|---|
| | | Univariable | | | Multivariable (n=241) | | |
| Feature | n | HR | 95%(CI) | p | HR | 95%(CI) | p |
| STR | 55/299 | 1.41 | 0.85-2.33 | 0.18 | 1.20 | 0.66-2.19 | 0.55 |
| M+ | 77/298 | 2.95 | 1.92-4.55 | < 0.0001 | 3.18 | 1.89-5.32 | < 0.0001 |
| LCA | 38/256 | 2.19 | 1.25-3.83 | 0.0059 | 2.41 | 1.29-4.48 | 0.0056 |
| *MYCN* | 22/277 | 2.349 | 1.24-4.47 | 0.0092 | 1.66 | 0.81-4.00 | 0.17 |

**Supplementary Table 3**

| | | Non-CSI treated patients | | | | | |
|---|---|---|---|---|---|---|---|
| | | Univariable | | | Multivariable (n=100) | | |
| Feature | n | HR | 95%(CI) | p | HR | 95%(CI) | p |
| STR | 30/114 | 1.54 | 0.85-2.81 | 0.15 | 1.83 | 0.90-3.72 | 0.095 |
| M+ | 36/113 | 1.59 | 0.90-2.78 | 0.11 | 1.26 | 0.68-2.34 | 0.46 |
| LCA | 14/93 | 7.06 | 3.62-13.79 | < 0.0001 | 3.29 | 1.36-7.96 | 0.0082 |
| *MYC* | 11/108 | 9.39 | 4.47-19.71 | < 0.0001 | | - | |

## Supplementary Table 4

| | | CSI treated patients | | | | | | | | | | | |
|---|---|---|---|---|---|---|---|---|---|---|---|---|---|
| | | Univariable | | | Univariable (imputed n=299) | | | Multivariable (n=241) | | | Multivariable (imputed n=299) | | |
| Feature | n | HR | 95%(CI) | p | HR | 95%(CI) | p | HR | 95%(CI) | p | HR | 95%(CI) | p |
| STR | 55/299 | 1.41 | 0.85-2.33 | 0.18 | 1.41 | 0.85-2.35 | 0.18 | 1.20 | 0.66-2.19 | 0.55 | 1.15 | 0.67-1.98 | 0.61 |
| M+ | 77/298 | **2.95** | **1.92-4.55** | **< 0.0001** | **2.94** | **1.89-4.57** | **<0.0001** | **3.18** | **1.89-5.32** | **< 0.0001** | **3.02** | **1.90-4.80** | **< 0.0001** |
| LCA | 38/256 | **2.19** | **1.25-3.83** | **0.0059** | **2.17** | **1.21-3.88** | **0.010** | **2.41** | **1.29-4.48** | **0.0056** | **2.24** | **1.18-4.26** | **0.015** |
| *MYCN* | 22/277 | **2.35** | **1.24-4.47** | **0.0092** | **2.25** | **1.16-4.35** | **0.017** | 1.66 | 0.81-4.00 | 0.17 | 1.64 | 0.78-3.46 | 0.19 |

## Supplementary Table 5

| | | Non-CSI treated patients | | | | | | | | | | | |
|---|---|---|---|---|---|---|---|---|---|---|---|---|---|
| | | Univariable | | | Univariable (imputed n=114) | | | Multivariable (n=100) | | | Multivariable (imputed n=114) | | |
| Feature | n | HR | 95%(CI) | p | HR | 95%(CI) | p | HR | 95%(CI) | p | HR | 95%(CI) | p |
| STR | 30/114 | 1.55 | 0.85-2.81 | 0.15 | 1.55 | 0.85-2.85 | 0.15 | 1.83 | 0.90-3.72 | 0.095 | 1.74 | 0.87-3.45 | 0.11 |
| M+ | 36/113 | 1.59 | 0.90-2.78 | 0.11 | 1.60 | 0.89-2.85 | 0.11 | 1.26 | 0.68-2.34 | 0.46 | 1.45 | 0.79-2.66 | 0.23 |
| LCA | 14/93 | **7.06** | **3.62-13.79** | **< 0.0001** | **6.66** | **3.17-14.00** | **< 0.0001** | **3.29** | **1.36-7.96** | **0.0082** | **3.49** | **1.37-8.78** | **0.010** |
| *MYC* | 11/108 | **9.39** | **4.47-19.71** | **< 0.0001** | **8.95** | **4.17-19.21** | **< 0.0001** | - | | | | | |

## Supplementary Table 6

| Subcontext | | n | Whole cohort - specific disease context univariable analysis (STR vs GTR) | | |
|---|---|---|---|---|---|
| | | | HR | 95%(CI) | p |
| All patients | | | | | |
| | CSI | 127/784 | 1.65 | 1.21-2.25 | 0.001 |
| | Non-CSI | 64/258 | 1.27 | 0.84-1.93 | 0.26 |
| M+ | | | | | |
| | CSI | 60/231 | 1.31 | 0.87-1.99 | 0.20 |
| | Non-CSI | 26/76 | 1.36 | 0.74-2.50 | 0.32 |
| M0 | | | | | |
| | CSI | 65/539 | 1.46 | 0.90-2.36 | 0.13 |
| | Non-CSI | 35/163 | 1.01 | 0.54-1.91 | 0.97 |
| CTX | | | | | |
| | CSI | 122/744 | 1.49 | 1.08-2.05 | 0.016 |
| | Non-CSI | 58/237 | 1.23 | 0.79-1.92 | 0.35 |
| WNT | | | | | |
| | CSI | 7/83 | 9.69 | 0.60-155.20 | 0.11 |
| | Non-CSI | | | na | |
| SHH | | | | | |
| | CSI | 20/130 | 1.77 | 0.87-3.62 | 0.12 |
| | Non-CSI | 31/127 | 1.47 | 0.75-2.88 | 0.26 |
| Group 3 | | | | | |
| | CSI | 22/157 | 1.87 | 1.03-3.38 | 0.039 |
| | Non-CSI | 19/74 | 0.68 | 0.32-1.43 | 0.31 |
| Group 4 | | | | | |
| | CSI | 69/358 | 1.39 | 0.67-2.22 | 0.17 |
| | Non-CSI | 6/33 | 2.01 | 0.55-7.83 | 0.28 |

## Supplementary Table 7

| Subcontext | | n | UK cohort - specific disease context univariable analysis (STR vs GTR) | | |
|---|---|---|---|---|---|
| | | | HR | 95%(CI) | p |
| All patients | | | | | |
| | CSI | 55/299 | 1.41 | 0.85-2.33 | 0.18 |
| | Non-CSI | 19/65 | 1.77 | 0.88-3.55 | 0.11 |
| M+ | | | | | |
| | CSI | 25/75 | 0.90 | 0.45-1.79 | 0.77 |
| | Non-CSI | 9/29 | 2.22 | 0.86-5.73 | 0.098 |
| M0 | | | | | |
| | CSI | 30/221 | 1.28 | 0.60-2.75 | 0.52 |
| | Non-CSI | 10/26 | 1.29 | 0.45-3.71 | 0.64 |
| CTX | | | | | |
| | CSI | 53/291 | 1.29 | 0.76-2.17 | 0.35 |
| | Non-CSI | 17/60 | 1.89 | 0.91-3.91 | 0.087 |
| SHH | | | | | |
| | CSI | 6/38 | 2.86 | 0.75-10.84 | 0.12 |
| | Non-CSI | 5/29 | 7.52 | 2.03-27.87 | 0.0025 |
| Group 3 | | | | | |
| | CSI | 8/50 | 1.66 | 0.60-4.54 | 0.33 |
| | Non-CSI | 6/22 | 0.49 | 0.14-1.76 | 0.28 |
| Group 4 | | | | | |
| | CSI | 31/128 | 1.02 | 0.46-2.26 | 0.96 |
| | Non-CSI | | | na | |
